# Simple Display System of Mechanical Properties of Cells and Their Dispersion

**DOI:** 10.1371/journal.pone.0034305

**Published:** 2012-03-30

**Authors:** Yuji Shimizu, Takanori Kihara, Seyed Mohammad Ali Haghparast, Shunsuke Yuba, Jun Miyake

**Affiliations:** 1 Department of Mechanical Science and Bioengineering, Graduate School of Engineering Science, Osaka University, Toyonaka, Osaka, Japan; 2 Health Research Institute, National Institute of Advanced Industrial Science and Technology (AIST), Amagasaki, Hyogo, Japan; Dalhousie University, Canada

## Abstract

The mechanical properties of cells are unique indicators of their states and functions. Though, it is difficult to recognize the degrees of mechanical properties, due to small size of the cell and broad distribution of the mechanical properties. Here, we developed a simple virtual reality system for presenting the mechanical properties of cells and their dispersion using a haptic device and a PC. This system simulates atomic force microscopy (AFM) nanoindentation experiments for floating cells in virtual environments. An operator can virtually position the AFM spherical probe over a round cell with the haptic handle on the PC monitor and feel the force interaction. The Young's modulus of mesenchymal stem cells and HEK293 cells in the floating state was measured by AFM. The distribution of the Young's modulus of these cells was broad, and the distribution complied with a log-normal pattern. To represent the mechanical properties together with the cell variance, we used log-normal distribution-dependent random number determined by the mode and variance values of the Young's modulus of these cells. The represented Young's modulus was determined for each touching event of the probe surface and the cell object, and the haptic device-generating force was calculated using a Hertz model corresponding to the indentation depth and the fixed Young's modulus value. Using this system, we can feel the mechanical properties and their dispersion in each cell type in real time. This system will help us not only recognize the degrees of mechanical properties of diverse cells but also share them with others.

## Introduction

The primary method of material recognition by humans is visualization, and other available methods are contact force and tactile sense (i.e., haptics). In particular, in the recognition of material that is not directly visible, haptics provides unique information. Furthermore, haptics is the fundamental nature of material recognition in all forms of life. The information of the haptics of a given material is one of the important tools for communication and sharing the features of the material.

The mechanical properties of biological cells are unique indicators of their states. Malignant cancer cells exhibit lesser stiffness than normal cells [1]. Red blood cells infected with *Plasmodium falciparum* exhibit higher stiffness than uninfected cells [2]. In optic-cup morphogenesis, the stiffness alterations of the retinal epithelium are important for the self-formation of neural retina tissue [3]. Furthermore, the information of the stiffness of mesenchymal stem cells (MSCs) is related to their diverse characters and states [4]–[6]. The reasons for the importance of the mechanical properties of cells are that the mechanical properties are largely determined by the actin cytoskeleton [6]–[10]. The actin cytoskeleton is the essential element for regulating cell function [11]–[13].

Several techniques have been successfully employed to study the mechanical properties of cells, including micropipette aspiration, magnetic twisting cytometry, optical traps, and atomic force microscopy (AFM) [14]–[16]. The latter method can be used to image live cells and probe their mechanical properties in physiological conditions in a nondestructive manner and at a high spatial resolution [17], [18]. It analyzes the mechanical properties of a living cell by the probe indentation method [7], [19] and force modulation method [20]. In the probe indentation method, which is viscerally and easily comprehensible, an AFM cantilever serves as a microindenter to probe the cell directly.

Although the mechanical properties of a cell can be examined by using these methods, it is difficult to perceive the mechanical properties of cells and their cell type-specific differences, because the size of a cell is too small and the values of the mechanical properties of a cell exhibit very broad distributions, even for the same point on a cell [6]. It is also difficult to communicate or share the mechanical information of a cell with other people based on only numerical values. If the mechanical properties of a cell can be perceived via a system, then these issues can be resolved and many people will understand the mechanical information of a cell, thereby stimulating the research field of cell mechanics. Moreover, we can comprehend the mechanical interaction between 2 adjacent cells and between a cell and its physical microenvironment.

The mechanical properties of the organs and tissues of humans can be displayed by virtual reality techniques [21], [22]. In particular, stiffness and force interactions are demonstrated by using a haptic device, which gives the operator force feedback directly [23]–[25]. Ladjal et al. used a haptic device to display the cell indentation process and simulate AFM experiments [26], [27]. They developed a computer-based training system to simulate real-time ES cell indentation procedures in virtual environments through the combination of a haptic device and finite element simulations [27]. Thus, haptic devices are very useful communication tools for sharing the mechanical properties and information of a cell.

In this study, we constructed a simple virtual reality system using a haptic device that displays the mechanical properties of cells as determined by AFM nanoindentation experiments. To homogeneously display various types of cell morphology in this system, we measured and presented the mechanical properties of cells at floating state, because the morphology of floating cell is almost spherical irrespective of cell type. This system virtually displays the dispersion of the Young's modulus of a cell and the differences in Young's modulus between cell types. Using this system, we can directly recognize the mechanical information of a cell and share its perceived properties with others.

## Materials and Methods

### Materials

A tipless probe (TL-CONT; spring constant: 0.03 N/m) was purchased from Nanosensors (Neuchatel, Switzerland). Cell culture medium was purchased from Nacalai Tesque (Kyoto, Japan), and fetal bovine serum (FBS) was purchased from JRH Biosciences (Lenexa, KS). Antibiotics were purchased from Sigma-Aldrich (St. Louis, MO). Human bone marrow-derived mesenchymal stem cells (hMSCs) were obtained from a donor (age, 39 years) with written informed consent as previously described [28]. The study was approved by the ethics committee of National Institute of Advanced Industrial Science and Technology. HEK293 cells were obtained from Health Science Research Resources Bank (Osaka, Japan). Biocompatible Anchor for Membrane (BAM; SUNBRIGHT OE-020CS) was purchased from NOF CORPORATION (Tokyo, Japan). Spherical silica beads (4 µm in diameter) were purchased from Ube-Nitto Kasei (Tokyo, Japan). A haptic device, Falcon, was purchased from Novint Technologies Inc (Albuquerque, NM). A PC, the performance of which is shown in Table 1, was purchased from Sony corporation (Tokyo, Japan). Other reagents were obtained from Sigma-Aldrich, Wako Pure Chemical Industries Ltd. (Osaka, Japan), or Life Technologies Japan Ltd. (Tokyo, Japan).

**Table 1:** System performance.

CPU	Intel core i3 (2.53 GHz)
Main memory	4.0 GB
OS	Microsoft Windows 7
Development environment	Microsoft Visual Studio 2010
Haptic device	Novint Falcon

### Preparation of BAM-coated dishes

**Figure 1:**
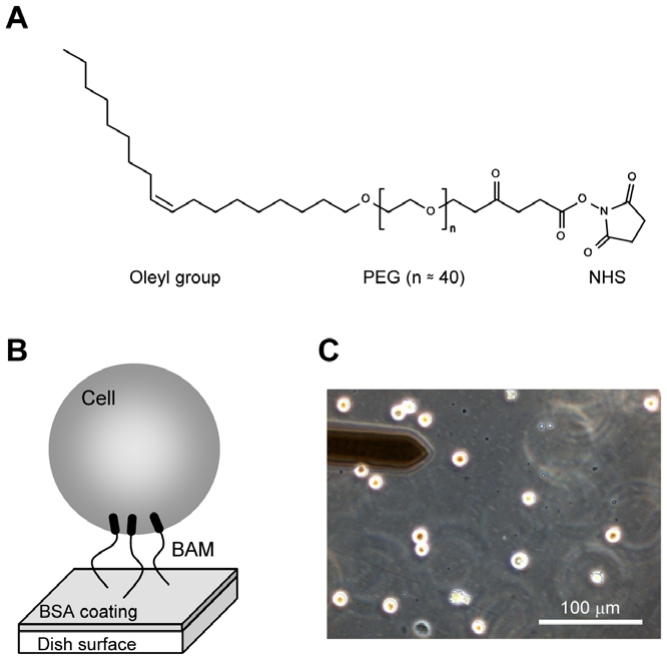
The Biocompatible Anchor for Membrane (BAM) system. (A) Chemical structure of BAM. It comprises an oleyl group, an NHS reactive ester group, and a hydrophilic PEG linker. (B) Diagram of the BAM-coated substrate. BAM molecules are fixed on the BSA-coated substrate via coupling with the NHS ester of BAM to the amino group of BSA. The surface oleyl group enters the plasma membrane of the cell. Then, the cell is anchored onto the BAM substrate. (C) Phase contrast micrograph of hMSCs on the BAM substrate. The floating cells were anchored to the substrate, maintaining their round shape. The left upper object is the AFM cantilever.

The BAM-coated dishes (Fig. 1) were prepared as described previously with minor modifications [29]. Briefly, polystyrene tissue culture dishes were coated with 5% BSA in PBS for 1 h. After washing with PBS, the surfaces were treated with 1 mM BAM in PBS for 1 h. Then, the BAM-coated dishes were washed and dried.

### Cell cultures

hMSCs were maintained in alpha-MEM containing 15% FBS and antibiotics (100 units/mL penicillin G, 100 µg/mL streptomycin sulfate, and 0.25 µg/mL amphotericin B). HEK293 cells were maintained in DMEM containing 10% FBS and antibiotics. The culture medium was replaced 2 or 3 times a week. Cells were removed from the culture dish by treatment with 0.25% trypsin-0.02% EDTA in PBS and then plated on the BAM-coated dish for 30 min in culture medium. The cells attached to the BAM surface were manipulated by AFM (Fig. 1C). The cell diameter was measured from the microscopic image of the cell attached to the BAM surface.

### AFM measurements

**Figure 2:**
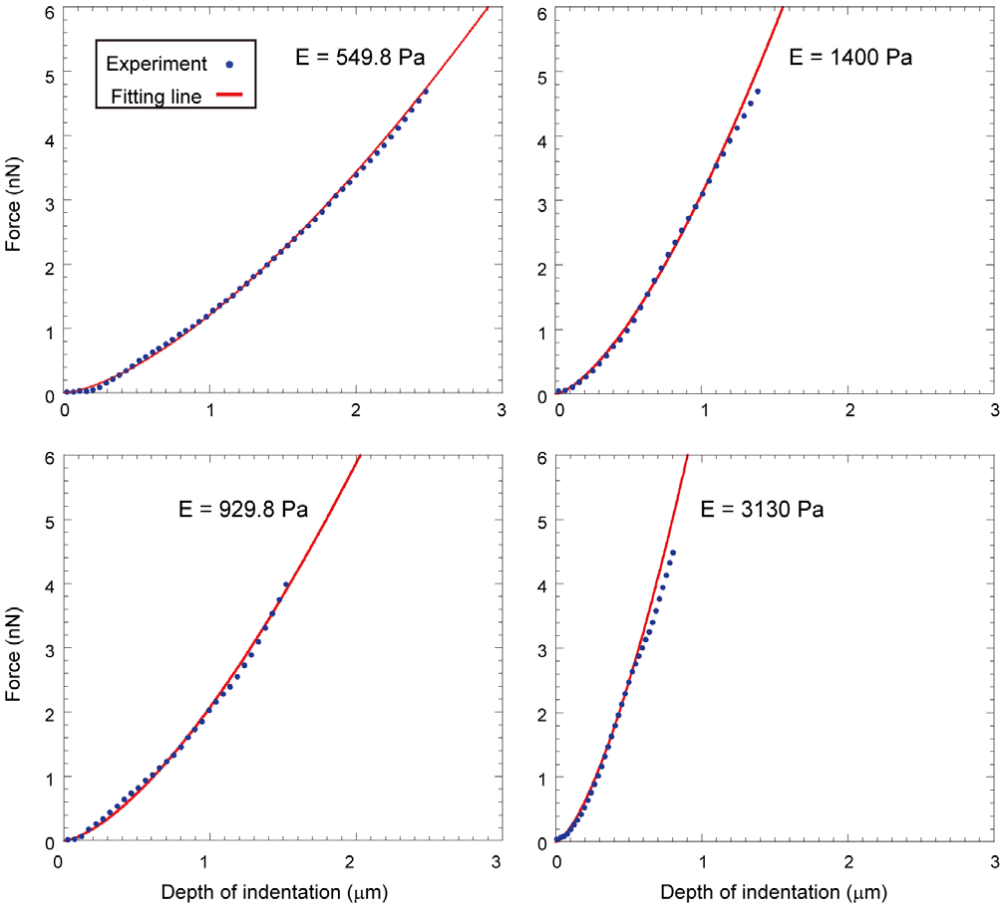
Typical force-distance curves obtained from the AFM indentation experiments using hMSCs. The blue points show experimental force curve lines, and the red line shows the Hertz model fitting line. The force curve at the region up to approximately 2 nN of cell surface indentation was fitted by the Hertz model. The fitting lines were well fitted to the experimental force curves in all experimental ranges.

The spherical silica probe was made by bonding the silica bead onto the edge of the tipless probe with epoxy resin. hMSCs and HEK293 cells attached to the BAM-coated dishes in the medium were manipulated by AFM (Nanowizard I, JPK Instruments AG, Berlin, Germany) at room temperature. The probe indented the top of the cells up to 5 nN at 10 µm/s. The Young's modulus of the cell was calculated in accordance with the Hertz model [30]. The force-distance curve at the region up to approximately 2 nN of the cell surface indentation was fitted by JPK data processing software (JPK instruments AG) as follows:
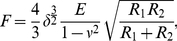
(1)where *F* = force, *δ* = depth of indentation, *R_1_* = radius of spherical probe (2 µm), *R_2_* = radius of the cell (cell type-dependent), *ν* = Poisson's ratio (0.5), and *E* = Young's modulus (Fig. 2).

All experiments were performed using more than 100 cells, and each cell was examined at more than 25 points on the top of the cell. The median value was adopted for the Young's modulus of each cell [6]. The Young's modulus of each cell was plotted in a log histogram. The histogram was fitted by a log-normal distribution, the equation for which is as follows:
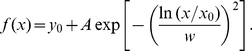
(2)The mode (*x_0_*) and variance parameter *w* were obtained from the fitting.

### Virtual reality system

**Figure 3:**
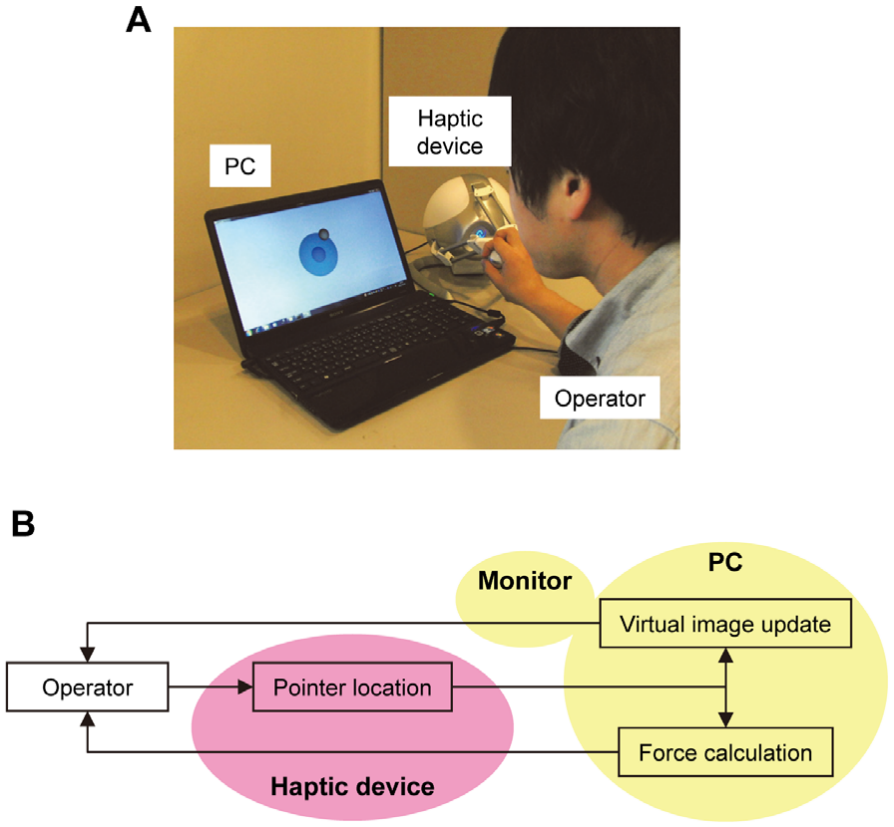
Virtual reality system. (A) Photograph of the system operation. The operator moves the handle of the haptic device while looking at the virtual image of the probe indentation process on the PC monitor. The operator feels the feedback force via the haptic device in conjunction with the virtual probe indentation to the cell object. (B) The relationship of the system components.

**Figure 4:**
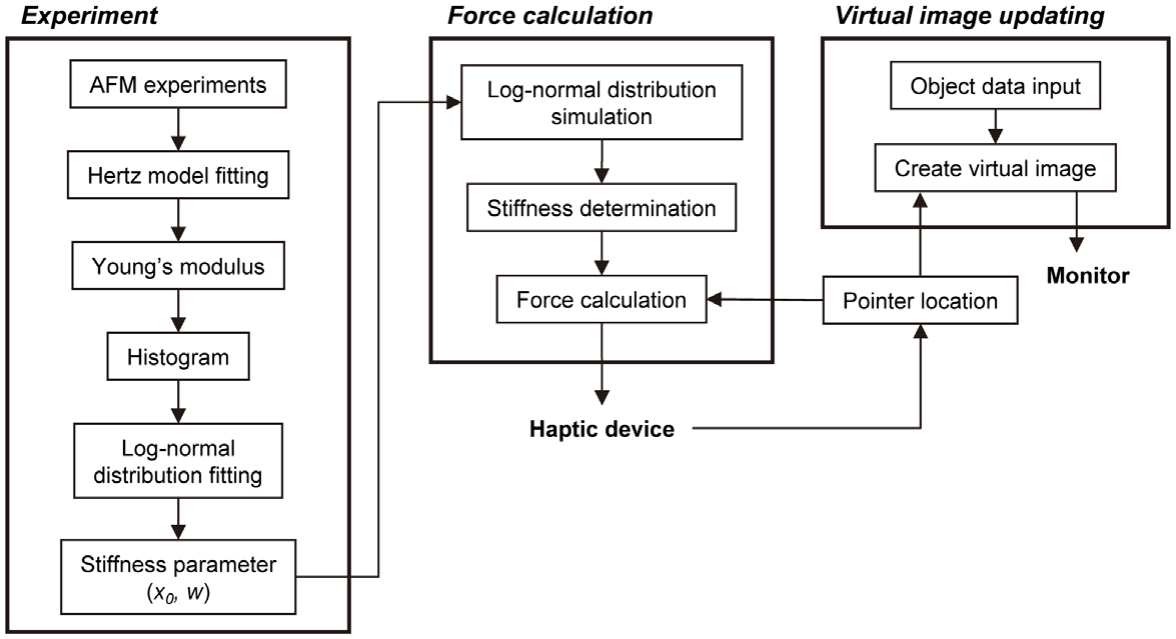
Detailed workflow of our system. There are 3 parts: experiment, force calculation, and virtual image updating. The stiffness parameters were previously obtained via experimentation. The obtained stiffness parameters were used in the force calculation, and the information about the cell diameter was used for virtual image updating.

Fig. 3A shows an image of the virtual reality system of cell indentation manipulation. An operator can feel and observe cell mechanics by moving the handle of the haptic device and touching the virtual cell object with the virtual spherical probe on the PC monitor (Fig. 3A). Virtual image updating and force calculation are performed automatically in a real-time manner with pointer locomotion. Then, the updated virtual image is transferred to the PC monitor, and the calculated force feedback is returned to the operator via the haptic device (Fig. 3). The detailed workflow, including experiments, force calculation, and virtual image update is shown in Fig. 4.

### Virtual image

**Figure 5:**
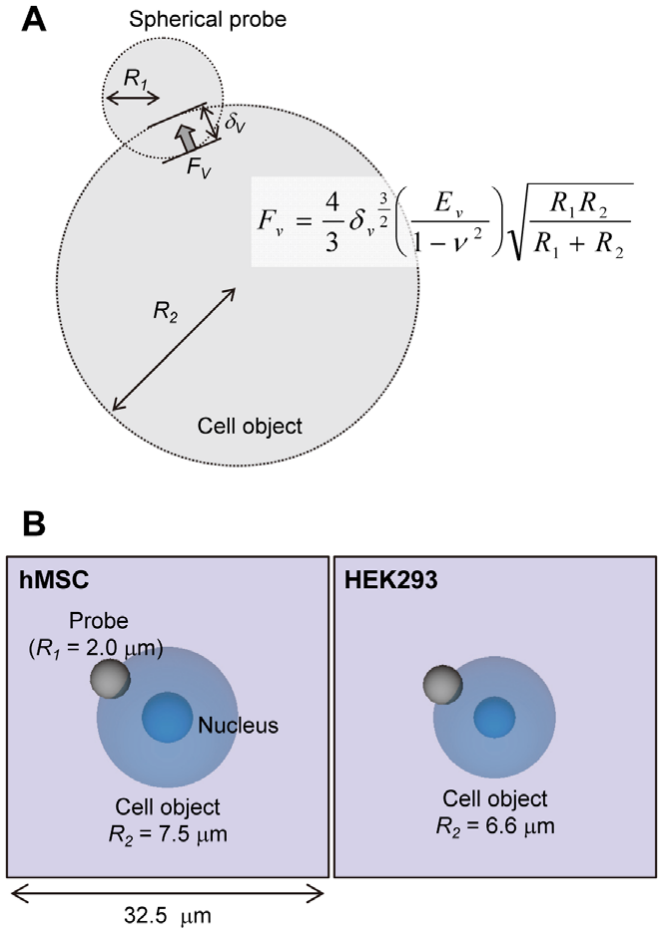
Virtual image of probe indentation in the cell. (A) Model of the cell object and the probe. The cell object and the probe were modeled as simplified spherical shapes. *R_1_* and *R_2_* are the radii of the probe and the cell object, respectively. *δ_v_* is the indentation depth of the probe into the cell object. The push back force (*F_v_*) to the probe from the cell was calculated by the Hertz model using *δ_v_* and the determined *E_v_*. (B) The representative virtual images. The left image represents hMSCs, and the right image represents HEK293 cells. The cell radii (*R_2_*) of hMSCs and HEK293 cells were 7.5 and 6.6 µm, respectively. The blue nucleus is fixed in the center of the cell object. The virtual space is 32.5 µm×32.5 µm×32.5 µm. The probe can be moved freely in the virtual space.

The cell and the probe were modeled as simplified sphere shapes and represented as spherical objects (Fig. 5A). The nucleus was set as the center of the cell object (Fig. 5B). The relative diameter of the cell in the virtual image could be changed by altering the cell diameter. The virtual space was set as 32.5 µm×32.5 µm×32.5 µm (Fig. 5B). The cell object was immobilized in the center of the virtual space, and the operator moved the probe object by manipulation with the handle of the haptic device. The cell and the probe object were only used in displaying the physical relationship in the virtual image, and thus, the cell object did not display the deformation caused by probe indentation (Fig. 5B).

### Haptic representation

The motion space of the haptic handle is 101 mm×101 mm×101 mm in real space. The virtual cell stiffness is represented by the Hertz model using the virtual depth of indentation (*δ_v_*) of the probe to the cell object (Fig. 5A). The stiffness parameters, the mode (*x_0_*) and the variance (*w*) of the log-normal fitted distribution of the Young's modulus, were introduced into the simulation program, and the represented stiffness (Young's modulus, *E_v_*) was determined with each contact of the probe and the cell objects by the Box-Muller method [31], which creates log-normal distribution-dependent random numbers by using these stiffness parameters. The details are as follows. The 2 independent random variables, α and β, are uniformly distributed in the interval (0, 1). The random variable *N* with a normal distribution of standard deviation 1 is given as
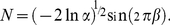
(3)Then, using *N*, the *E_v_* is given as
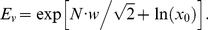
(4)Finally, the represented *F_v_*, which was determined by a Hertz model equation with the determined *E_v_* and *δ_v_*, is sent to the haptic device in real time.

## Results and Discussion

### Experimental cell stiffness

Many types of cells are used for cell culture. Thus, innumerable cell types were available for use in evaluating the mechanical properties of a cell by the virtual reality system. The mechanical properties of a cell are represented using the haptic device, and the physical relationship between the cell and the probe is represented on the PC monitor by displaying these findings virtually. On the other hand, cultured cell morphology is different in each cell and each cell type. Thus, to display the morphology of all types of cells easily, we represented the mechanical properties in the floating cell state in our virtual reality system. The morphology of the floating cell is almost spherical irrespective of cell types, and it is easy to display the spherical object by using a PC.

The mechanical properties of surface-immoliblized nonadherent leukocytes using AFM have been reported earlier [10], [32]. Here, we used the BAM substrate to determine the mechanical properties of floating cells by AFM. BAM contains a cytoplasmic anchoring oleyl group, a hydrophilic PEG domain, and an NHS reactive ester group [29]. The BAM substrate was constructed from the BSA substrate by coupling the NHS ester of the BAM molecule to the amino group of BSA (Fig. 1B). The trypsinized floating cell attached to the BAM substrate spontaneously (Fig. 1C). The attached cell was anchored to the substrate tightly, and it was not removed from the surface by swinging. Then, we measured cell stiffness in the floating state by the probe indentation method using AFM (Fig. 1C).

Fig. 2 shows the typical force curves and their Hertz model fitting curves in hMSC experiments. Although the adaptation range of the Hertz model is limited by the small indentation depth of the probe, the fitting curves were well fitted in the entire experimental indentation range (Fig. 2). The reasons for the good fitting are the isotropy of the floating cell on the BAM substrate and the simple indentation process of the 2 spherical objects (spherical cell and probe). An adhered cell has anisotropic membrane tension and actin fibers [33]. Furthermore, the shape and the structure of an adhered cell are complicated and varied. These elements complicate the indentation process of the probe to the cell surface. In this study, the represented force *F_v_* corresponding to the indentation depth of the probe was calculated with the Hertz model equation (Fig. 5).

**Figure 6:**
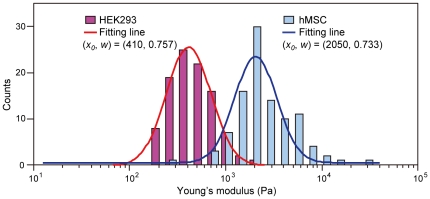
Histogram of the Young's modulus distributions of hMSCs and HEK293 cells. Each histogram consists of the Young's modulus data of 100 cells. Each histogram is fitted with a log-normal pattern (fitted line). The fitting stiffness parameters, mode value *x_0_*, and variance parameter *w* are shown.

Fig. 6 shows histograms of the Young's modulus of hMSCs and HEK293 cells. The distributions of Young's modulus of these cells were clearly different, and the Young's modulus of hMSCs was higher than that of HEK293 cells (Fig. 6). The average cell diameters of hMSCs and HEK293 cells were 15.0±3.0 and 13.2±2.2 µm, respectively. The Young's modulus of each cell was distributed broadly and in each range was extended by single digits for both cell types (Fig. 6). Then, each Young's modulus distribution was fitted by a log-normal pattern. The mode values (*x_0_*) of the Young's modulus of hMSCs and HEK293 cells were 2050 and 410 Pa, respectively. The variance parameters *w* for these cells were 0.733 and 0.757, respectively. Therefore, the breadth of the distribution of Young's modulus was similar in both cells in the floating state. It is, however, unclear whether the breadth of the Young's modulus distribution of the floating cell is almost identical.

**Figure 7:**
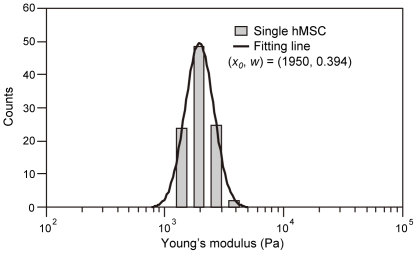
Histogram of the Young's modulus distribution of a single hMSC measurement. A single hMSC was measured 100 times. The histogram was fitted with a log-normal pattern.

In contrast, the distribution of the Young's modulus of a single cell was narrower than that of the same cell type (Fig. 7). The mode value (*x_0_*) and variance parameter *w* of the log-normal fitted distribution of Young's modulus of a single hMSC were 1950 Pa and 0.394 respectively. In this study, we developed a virtual reality system to represent the mechanical properties (Young's modulus and its variance) of each cell type.

### Virtual reality system

**Figure 8:**
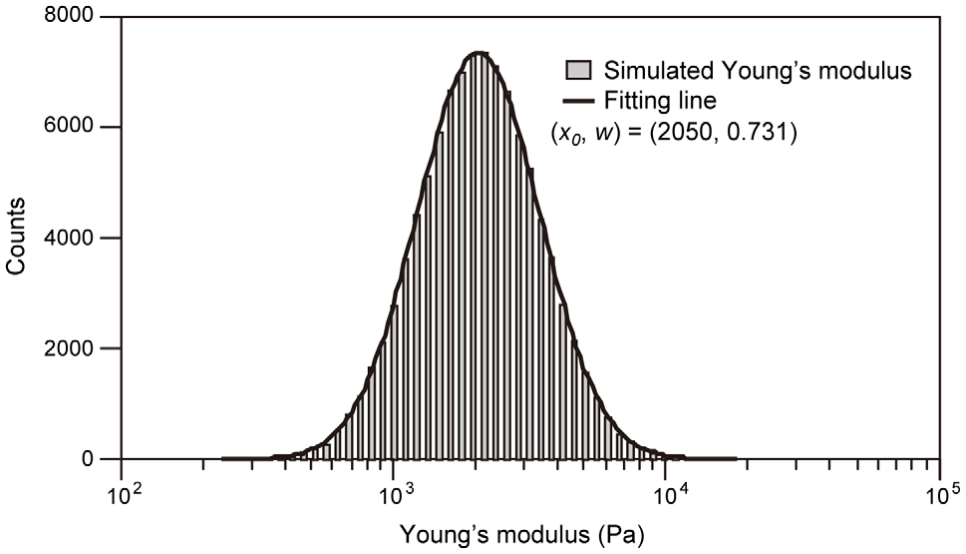
Histogram of the simulated Young's modulus distribution obtained using the Box-Muller method. The simulation was trialed 100,000 times using hMSC stiffness parameters. The histogram was fitted with a log-normal pattern. The represented Young's modulus (*E_v_*) was in accordance with the simulated value.

In the virtual reality system, to represent the aforementioned mechanical properties of the cell, we adopted the Box-Muller method for determining Young's modulus, which creates log-normal distribution-dependent random numbers [31]. Fig. 8 shows the distribution of the simulated values by the Box-Muller method using the parameters of the mechanical properties of hMSCs. Using this method, we succeeded in demonstrating the log-normal distribution of the mechanical properties of hMSCs. The represented Young's modulus value was calculated for each touching event of the probe surface and the cell object (Fig. 5). The haptic presentation force (*F_v_*) was calculated regarding to the indentation depth (*δ_v_*) and the determined Young's modulus value (*E_v_*) (Fig. 5).

In presenting the calculated force (*F_v_*) via the haptic device, its scale became controversial in real space. The scale of the calculated force (*F_v_*) was almost in the range of pN to nN, and the value was too small to present the force in the haptic device. Even if considering the scale conversion ratio of real space to virtual space (101 mm in real space to 32.5 µm in virtual space), the scale of the force was almost in the range of µN (approximately 1×10^5^-fold more than the calculated *F_d_*). Therefore, we prioritized the recognition of the relation and difference of the mechanical properties of each cell type in real space rather than presenting the real value. The presented force was enlarged 5×10^10^-fold in real space.

The spherical probe was subjected to drag force and buoyant force from the water and gravity in the background. The viscous drag *F_d_* for the spherical probe (*r* = 2 µm; *v* = 10 µm/s) is 0.337 pN at 25°C (Text S1). The gravity (*F_g_*) for the silica probe (*ρ* = 2200 kg/m^3^) is 0.723 pN, and the buoyant force (*F_b_*) is 0.329 pN (Text S1). These forces are much smaller than the *F_v_*, and thus, we did not account for their effects in our study.

On our PC, the performance of which is shown in Table 1, the time delay from the input to the output of the haptic device was ∼1 ms. The time interval required to calculate each Young's modulus at the contact of the probe and the cell surface was nearly 0 ms. These time delays (total, ∼1 ms) were sufficiently fast for the time resolution of human haptic recognition (approximately several 100 Hz) [34]. Therefore, our virtual reality system succeeded in representing the mechanical properties of a cell in real time.

The supplemental video S1 shows the actual situation of the system operation. The depth of each probe indentation with constant force varied in each cell touching, and occasionally virtual cell showed very high or low stiffness. Moreover, the virtual hMSC and HEK293 cell apparently showed different stiffness. These differences among the same type of cells or between each cell type were clearly understood by using this virtual reality system.

Our system consisted of a PC, including a monitor, and the haptic device. The haptic device (Falcon), which is offered commercially as a PC game controller, is a low-budget device, and our PC is a common type. Moreover, our system does not require any special software or high performance computer due to excluding the display of cell deformation. Thus, our developed virtual reality system can be easily created in any laboratory and for any exhibition, and the only necessary information is the mechanical properties of a cell, i.e., the mode and variance of the distribution of the Young's modulus of the cell. These mechanical properties can be gathered in a database. In short, using our system, anyone can perceive the mechanical properties of any existing cell type. In the future, we expect that the mechanical properties of cells will become tangible features for sharing and communication with many people.

### Conclusions

This study introduced a new simple display system that presented the mechanical properties and their dispersion of each cell type as measured by AFM. The system enabled presentation of the mechanical properties of various floating cell types by simulating the AFM nanoindentation experiments. It is indeed difficult to imagine or viscerally understand the mechanical properties of cells and their cell type-specific differences, also the mechanical interaction between adjacent cells or between a cell and its physical microenvironment. Therefore, we are scarcely able to communicate or share the mechanical properties of cells with others. According to our developed mechanical information display system by using virtual reality technology, we can not only recognize the mechanical properties of each cell but also share them. This will make the mechanical information of cells an intelligible character for researchers, including biochemists, cell biologists, and developmental biologists, thereby stimulating the research field of cell mechanics.

## Supporting Information

Text S1 The physical forces for the AFM probe in water.
(PDF)

Video S1 Video imaging of the system operation. The first part of the video shows the situation of the operation for virtual hMSC, and the last part shows the operation for virtual HEK293 cell. Apparently, the lengths of the probe indentation vary between virtual hMSC and HEK293 cell.
(WMV)
