# Supplementary material for: Simple Display System of Mechanical Properties of Cells and Their Dispersion
Source: PLoS One. 2012 Mar 30;7(3):e34305. doi: 10.1371/journal.pone.0034305 (PMC3316616; doi:10.1371/journal.pone.0034305)
Supplement: Text S1 — The physical forces for the AFM probe in water. (PDF) [file pone.0034305.s001.pdf]

Supporting information

Text S1

The physical forces for the AFM probe in water are gravity (F_d), buoyant force (F_b), and viscous drag (F_d). These forces are determined as follows:

$$F_d = 6\pi r \eta v$$

$$F_g = \frac{4}{3}\pi r^3 \rho_s g$$

$$F_b = \frac{4}{3}\pi r^3 \rho_w g$$

where η = viscous coefficient of water, v = probe moving speed; ρ_s = silica density, ρ_w = water density, and g = acceleration of gravity.
